# Thirty Mouse Strain Survey of Voluntary Physical Activity and Energy Expenditure: Influence of Strain, Sex and Day–Night Variation

**DOI:** 10.3389/fnins.2020.00531

**Published:** 2020-07-07

**Authors:** Christine König, Anne-Christine Plank, Alexander Kapp, Ivanna K. Timotius, Stephan von Hörsten, Katharina Zimmermann

**Affiliations:** ^1^Department of Anesthesiology, Universitätsklinikum Erlangen, Friedrich-Alexander-Universität Erlangen-Nürnberg, Erlangen, Germany; ^2^Department of Experimental Therapy, Preclinical Experimental Center, Universitätsklinikum Erlangen, Friedrich-Alexander-Universität Erlangen-Nürnberg, Erlangen, Germany; ^3^Machine Learning & Data Analytics Lab, Department of Computer Science, Friedrich-Alexander-Universität Erlangen-Nürnberg, Erlangen, Germany; ^4^Department of Electronics Engineering, Satya Wacana Christian University, Salatiga, Indonesia

**Keywords:** respiratory exchange ratio (RER), sexual dimorphism, inbred mouse strains, indirect calorimetry assessment, phenotype screening

## Abstract

We measured indirect calorimetry and activity parameters, VO_2_ and VCO_2_ to extract respiratory exchange ratio (RER) and energy expenditure in both sexes of 30 inbred mouse strains of 6 genetic families at 9–13 weeks during one photophase and the subsequent scotophase. We observed a continuous distribution of all traits. While males had higher body weights than females, we observed no sex difference for food and water intake. All strains drank and fed more during the night even if they displayed no day–night difference in activity traits. Several strains showed absent or weak day–night variation in one or more activity traits and these included FVB and 129X1, males of 129S1, SWR, NZW, and SM, and females of SJL. In general females showed higher rearing and ambulatory activity with 6 and 9 strains, respectively, showing a sex difference. Fine motor movements, like grooming, showed less sex differences. RER underlied a strong day–night difference and no sex effect. Only FVB females and males of the RIIIS and SM strain had no day–night variation. Energy expenditure underlies a large day–night variation which was absent in SWR and in FVB females and RIIIS males. In general, female bodies had a tendency to higher energy expenditure values, which became a significant difference in C3H, MAMy, SM, DBA1, and BUB. Our data illustrate the diversity of these traits in male and female inbred mice and provide a resource in the selection of strains for future studies.

## Significance Statement

The use of inbred mouse strains in combination with genetic approaches has made an enormous contribution to our understanding of the genetic background of particular behavioral traits and diseases. Continuous assessment of the phenotypic diversity in inbred strains will therefore aid to the study of the physiology of particular traits and disease pathomechanisms.

## Introduction

Basic physiological and metabolic parameters are influenced by the genetic background and environmental factors and have a large impact on the outcome of behavioral and other experimental studies in mice. The majority of experimental animal studies in the past was based on the C57BL/6J mouse strain. In the last decades the availability of genetically diverse inbred mouse strains has increased, allowing the selection of specific inbred mouse strains for the investigation of the genetics of particular traits and for the development of suitable transgenic mouse models for diseases, provided that strain-specific information on relevant traits and physiological parameters are established. The information on phenotypic traits is constantly increasing and many of them are collected in the mouse phenome database^1^. Nevertheless, so far there is little comprehensive information on the genetic variability and on sex differences of the basal metabolic rate in combination with food and water intake and on home-cage activity at near-normal housing conditions. Since these parameters are of importance for the selection of inbred strains for studies related to obesity, nutrition, metabolism, locomotion and pharmacological drug dosing as well as for the development of suitable disease models, the goal of this study was to provide normative data of these parameters during day and nighttime and for both sexes. We screened male and female mice from 30 commonly available inbred mouse strains in a standardized metabolic cage system for ca. 48 h to extract one complete day–night cycle after at least 12 h of adaptation (one scotophase) in the home cage. Fully automated experimental setups help to eliminate confounding effects and support to establish strain-specific norms for other non-anesthetized and non-restrained murine models. They also add-on to classical mouse ethograms^2^. The strains in our study are representative for a wide range of genetic origins and belonged to 6 of the 7 mouse groups separated based on SNP analysis (Petkov et al., 2004): Bagg Albino Derivatives: A/J, AKR/J, Balb/cJ, C3H/HeJ, C3H/HeOuJ, CBA/J, LG/J, MRL/MpJ; Swiss mice: BUB/BnJ, FVB/NJ, MA/MyJ, NOD/ShiLtJ, RIIIS/J, SJL/J, SWR/J; Japanese and New Zealand inbred strains: KK/HlJ, NON/LtJ, NZB/BINJ, NZO/HlLtJ, NZW/LacJ; C57/58 strains: C57BL/6J, C57BL/6NJ, C57BL/6NCrl, Castle’s mice: 129S1/SvImJ, 129X1/SvJ, BTBR T Itpr3/J, LP/J; and the C. C. Little’s DBA and related strains: DBA/1J, DBA/2J, SM/J. Most of the strains are used in gene mapping and functional genetic analyses and rely on a valid and comprehensive characterization of behavioral traits including falsification and exclusion of potential confounding factors. Principally, variability of resting metabolic rate and physical activity may indirectly affect the readout of other phenotypic analysis. The availability of such data represents a valuable tool for experimental designing based on the selection of particular mouse strains and for correlation analysis.

## Materials and Methods

### Animals

Twenty-nine inbred mouse strains were purchased from the Jackson Laboratories (Bar Harbor, ME, United States) and one strain from Charles River (Sulzfeld, Germany), and colonies were established via brother × sister or offspring × parent mating at the preclinical research center of the Universitätsklinikum Erlangen. We used, in most strains, 12 male and 12 female mice aged youngest 48 and oldest 101 days of the F1 and F2 generations. A narrower age range was not possible due to space restrictions. The occurrence of significant age differences between some groups were non-intentional and disclosed in the results. Age and body weight were distributed across the strains as summarized in Table 1. The mice were housed in sex- and strain-matched groups of at least two and maximal five animals, according to FELASA recommendations (Rehbinder et al., 1996; Nicklas et al., 2002). All mice were kept under a 12:12 h light:dark cycle regulated between 4:30 a.m. and 4:30 p.m. Ambient temperature and humidity were kept at 22 ± 2°C and 55 ± 10%, respectively. Food and ozonized tap water were available *ad libitum*. All research and animal care procedures were reviewed by the local animal ethics committee (University of Erlangen) and approved by the local district government (Regierung von Unterfranken) under registry 55.2 2532-2-240. Experiments were conducted in accordance with the Guidelines of the European Parliament Council (directive 2010/62EU). The study conforms to the local as well as ARRIVE (Animal Research Reporting of *In Vivo* Experiments) guidelines (Kilkenny et al., 2010).

**TABLE 1:** Thirty inbred strains were screened in metabolic cages.

**Strain**	**Abbreviation**	**Stock #**	**Sex**	***N***	**Generation**	**Age (days)**	**Weight (g)**
129S1/SvImJ	129S1	002448	Male	10	F1	63.60 ± 1.71	24.11 ± 0.92
			Female	12	F1	73.92 ± 8.64	20.24 ± 2.36
129X1/SvJ	129X1	000651	Male	8	F1	65.25 ± 6.45	22.72 ± 1.26
			Female	6	F1	66.00 ± 7,67	18.50 ± 1.16
A/J	A	000646	Male	12	F1	68.67 ± 9.76	24.18 ± 2.00
			Female	12	F1, F2	65.33 ± 7.81	20.15 ± 2.34
AKR/J	AKR	000648	Male	11	F1	70.27 ± 5.61	28.40 ± 0.75
			Female	12	F1	72.50 ± 3.80	24.58 ± 1.56
Balb/cJ	Balb	000651	Male	11	F1, F2	81.27 ± 3.20	27.14 ± 1.14
			Female	11	F1, F2	77.27 ± 7.60	20.35 ± 1.08
BTBR T Itpr3/J	BTBR	002282	Male	12	F1	81.27 ± 7.02	31.20 ± 2.70
			Female	12	F1	83.00 ± 8.14	28.33 ± 1.98
BUB/BnJ	BUB	000653	Male	11	F1	66.64 ± 2.91	29.91 ± 1.99
			Female	11	F1	63.82 ± 6.35	24.77 ± 1.52
C3H/HeJ	C3H	000359	Male	12	F1	70.17 ± 1.64	25.08 ± 1.83
			Female	12	F1	68.42 ± 5.18	19.29 ± 0.91
C3H/HeOuJ	C3HOu	000635	Male	12	F1, F2	70.00 ± 2.22	27.9 ± 1.73
			Female	12	F1, F2	69.33 ± 0.49	22.85 ± 1.80
C57BL/6J	C57J	000664	Male	12	F1	79.17 ± 6.58	25.45 ± 1.29
			Female	12	F2	76.08 ± 3.92	20.56 ± 1.84
C57BL/6NCrl	C57NCrl		Male	12	F1, F2	79.17 ± 6.58	26.02 ± 2.56
			Female	12	F2	70.33 ± 6.61	20.18 ± 1.53
C57BL/6NJ	C57NJ	005304	Male	12	F1, F2	66.42 ± 3.48	26.48 ± 2.60
			Female	12	F1, F2	64.33 ± 4.33	19.47 ± 1.43
CBA/J	CBA	000656	Male	12	F1, F2	68.25 ± 8.24	24.81 ± 2.00
			Female	12	F1, F2	74.58 ± 8.78	22.44 ± 1.75
DBA/1J	DBA1	000670	Male	12	F1, F2	77.92 ± 9.11	21.31 ± 2.55
			Female	11	F2	69.00 ± 3.46	17.25 ± 2.39
DBA/2J	DBA2	000671	Male	11	F1	64.09 ± 1.04	24.53 ± 1.62
			Female	12	F1	64.67 ± 4.10	19.42 ± 1.36
FVB/NJ	FVB	001800	Male	12	F1	83.67 ± 4.92	30.00 ± 1.94
			Female	12	F1	77.45 ± 2.30	22.71 ± 2.56
KK/HlJ	KK	002106	Male	12	F1	71.92 ± 2.02	31.99 ± 2.43
			Female	12	F1	69.50 ± 7.14	30.10 ± 1.21
LG/J	LG	000675	Male	12	F1	72.83 ± 9.84	43.19 ± 4.14
			Female	12	F1	73.92 ± 8.08	37.91 ± 3.73
LP/J	LP	000676	Male	11	F1, F2	67.45 ± 7.61	21.35 ± 2.48
			Female	10	F1, F2	67.00 ± 5.79	16.89 ± 1.27
MA/MyJ	MAMy	000677	Male	9	F1, F2	61.00 ± 7.04	22.44 ± 1.09
			Female	10	F1	62.80 ± 10.7	18.82 ± 1.75
MRL/MpJ	MRL	000486	Male	12	F1	79.92 ± 1.88	45.39 ± 2.00
			Female	12	F1, F2	75.33 ± 3.60	35.13 ± 3.17
NOD/ShiLtJ	NOD	001976	Male	12	F1, F2	70.67 ± 7.38	25.20 ± 1.57
			Female	12	F1, F2	72.92 ± 8.90	21.56 ± 1.88
NON/LtJ	NON	002423	Male	12	F1	72.42 ± 2.07	33.17 ± 1.32
			Female	10	F1, F2	81.00 ± 4.74	27.99 ± 1.87
NZB/BINJ	NZB	000684	Male	12	F1	79.83 ± 3.97	29.33 ± 2.69
			Female	12	F1, F2	72.25 ± 10.10	24.48 ± 1.55
NZO/HlLtJ*	NZO	002105	Male	10	F1, F4	60.08 ± 7.55	46.33 ± 5.52
			Female	12	F1	73.67 ± 14.72	43.66 ± 7.95
NZW/LacJ	NZW	001058	Male	11	F1	89.55 ± 5.24	34.63 ± 1.83
			Female	12	F1	84.33 ± 9.54	30.10 ± 2.67
RIIIS/J	RIIIS	000683	Male	9	F1	92.33 ± 5.74	19.82 ± 2.03
			Female	11	F1	86.64 ± 11.37	15.43 ± 1.35
SJL/J	SJL	000686	Male	12	F1, F2	93.58 ± 2.27	25.71 ± 0.90
			Female	11	F1, F2	90.27 ± 2.72	21.48 ± 0.77
SM/J	SM	000687	Male	9	F1	82.44 ± 8.31	18.44 ± 3.45
			Female	9	F1	89.67 ± 2.50	16.22 ± 0.99
SWR/J	SWR	000689	Male	9	F1	88.11 ± 2.32	24.71 ± 2.06
			Female	9	F1, F2	77.78 ± 12.17	19.69 ± 0.73

The number of mice per sex, body weight and age are indicated in the table. Only mice from the F1 and F2 generations were included in the experiments and male and female mice per strain were adjusted to N(female) = N(male) ± ≤2. Age and weight data are shown as mean ± SD. * NZO/HiltJ males develop type 2 diabetes characterized by marked hyperglycaemia and hyperinsulinemia at 8–12 weeks (Joost and Schurmann, 2014). Females don’t develop type 2 diabetes.

### Experimental Design

Mice were monitored in the PhenoMaster automated home cage Phenotyping setup (TSE Systems, Germany) equipped with high-speed indirect calorimetry, as previously described (Chevessier et al., 2015). Experimental animals were transferred to the testing room at least 7 days before the beginning of the experiment and were kept in regular IVC Sealsafe Plus GM500 cages (Tecniplast S.p.A., Italy) in their usual environment with up to 4 littermates. The PhenoMaster experiments lasted 48 h and started latest at 4 p.m. with one scotophase, which served to adapt the mice to the environment of the newly provided home-cage within the PhenoMaster system/setup. The data acquired during the following day—night cycle were used for analysis. Light was generated by standard neon lights and, during the day, the intensity in the room was 1,000 lux and in the cage 400 lux. The experiment ended during the second day when a new group of mice was adapted in the cages.

### Automated Phenotyping Using Intra-Home-Cage Technology

The PhenoMaster system for mice automatically screens mice for several behavioral and metabolic parameters in a home-cage-like environment with a high temporal and spatial resolution. Data are continuously collected from single experimental animals under conditions that maximize the likelihood of natural behavior to take place, thus representing a refinement of the use of laboratory animals for research and delivering a “no-touch-ethogram” obtained from standardized experimental procedures.

The PhenoMaster measures indirect calorimetric parameters (Bode et al., 2009) and is based on conventional Sealsafe Plus GM500 cages equipped to individually monitor one mouse per cage. *Ad libitum* food and water intake are measured with specific sensors and cumulative consumption calculated by the system. A catch tray underneath the food hopper minimizes food spillage. Calorimetric parameters are assessed by continuously measuring the O_2_ and CO_2_ concentration in the cages through an open-circuit. The airtight PhenoMaster cage lids allows a stable airflow through the cage. Locomotor activity is measured by a lightbeam-based device, in which two infrared sensor frames lay on top of each other and surround the home cage. The lower one is fixed approximately 2 cm above the bedding (∼70 g of wood shavings, LTE E-001, ABEDD, Austria) and records horizontal locomotion of the mouse in *X*- and *Y*-plane (walking) whereas the upper one measures vertical movements (rearing) or exploration in *Z*-plane. Each frame contains sensor pairs with a beam wavelength of 950 nm that are arranged in strips for horizontal (*X* = 25 beams and *Y* = 16 beams) and vertical (*Z* = 25 beams) detection. Mouse movements or activity induced light beam breaks were counted in a user-defined time interval during the experimentally defined period of time (e.g., 48 h and light and night cycles, respectively). Analysis of these data was achieved by further sub-dividing beam-break counts into fine movements or stationary movements, like fidgeting or grooming (XF and YF), resulting from the repeated interruption of the same light beam and, ambulatory movements (XA and YA) which were counted as the consecutive interruption of different beams. The total number of *Z*-axis breaks was monitored and classified as rearing (Z). Only interruptions of the light beams classified as one of the above-mentioned behaviors were evaluated, so that minimal motions (such as breathing) were omitted. We analyzed physical activity as the cumulated total number of rearing events (Z) as well as of fine movements (XF + YF) and of ambulatory movements (XA + YA).

One pair of dedicated O_2_/CO_2_ sensors per cage enabled a continuous and simultaneous calculation of O_2_ consumption (VO_2_), CO_2_ production (VCO_2_), respiratory exchange ratio (RER) and energy expenditure (EE). Sensors were calibrated once a week with calibration gas mixtures (CO_2_, 0.05%, O_2_ 20.895% in N_2_; CO_2_ 0.950%, O_2_ 20.00%, in N_2_; both from Linde, Germany) and the sample airflow adjusted to 0.25l/min. The entire flow amounts 0.35l/min. The feeders were filled with standard laboratory chow V1124-300 (Sniff lab chow pellets; Germany) and water bottles were filled with ozonized tap water, obtained values are indicated as “Feeding” and “Drinking.” The cage temperature (Temp) was measured continuously via a high-end temperature sensor located inside the cage lid. Temp was previously identified to have a correlation with body temperature in the rat system (Urbach et al., 2014). Body weight (g) and age (days) of all mice was noted at the first experiment day. The animals were then placed in the cages and data acquired for up to 8 animals in parallel for the following 48 h. Disturbance during the testing period was kept to a minimum, entries into the room were recorded and the experimenter entered only once a day to control the PhenoMaster setup, inspect the status of the animals and refill water bottles/feeders, if necessary. After each experiment, all animals were returned to their original social groups, and transferred to the regular holding room.

### Data Mining and Analysis of Data Obtained With the PhenoMaster

All measurements were acquired using the PhenoMaster software, Version 4.8.9 (2013–4854), supplied by TSE. Raw data are compressed within the PM-system and can be exported with a time resolution defined by the experimenter. All variables were acquired at a resolution of one data point per minute. RER was calculated as the exhaled VCO_2_ (ml/h/kg) divided by the consumed VO_2_ (ml/h/kg) per min. Feeding (g) and drinking (ml) was derived as the amount of food and water consumed in total during the entire day (12 h) or night cycle (12 h). Whenever water bottles leaked, respective data were excluded from the analysis. The energy expenditure (EE) was acquired as kcal/h. For calibration reasons, calorimetric data of each cage were measured against an empty room temperature reference cage once in 3 h for 9 min. At these time intervals no values were acquired, and the missing data were not substituted, because the minute resolution was averaged to 20-min time intervals for further processing of data and figures.

### Data Analysis and Statistics

From the 20 min interval data, day and night means were calculated for each individual animal. Day and night means of each parameter were then subjected to an outlier analysis based on the 2.2-fold of the interquartile range, the IQR (Hoaglin and Iglewicz, 1987). The quartiles were calculated using weighted averages (SPSS) and the outliers were identified and removed with Excel. The heat maps were generated from effect sizes (see below) using R 3.5.1. The data were analyzed with SPSS. The groups for each of the eight traits (drinking, feeding, the three activity traits, cage temperature, RER, EE, all measured at day and night) consisted of 30 strains and were measured at two time points. These groups, including body mass, were separated by sex and subjected to the Kolmogorov–Smirnov normality test. Ninety one percent of the grouped data were normal distributed. Age was not expected to show a normal distribution. In the following, ANCOVA was considered robust enough to these few violations. Data mentioned in text refer to the respective values with the SD while figures illustrate error bars which represent the standard error of the mean.

(1) Day–night cycle, strain, sex effects, and interactions were assessed with repeated measures analysis of variance with covariates (RM-ANCOVA). First, main factors were day–night cycle (repeated-measures variable), strain and sex were between-subjects factors and, age and weight were registered as covariates (see Table 2). Effects are expressed as *F*-ratio and level of significance are indicated for each trait. Partial Eta squared $\eta_{\text{p}}^{2}$ was used to express how much of each factor contributed to the variance of each trait value and, to calculate the effect size *f*.

(2) Day–night cycle effects were assessed with RM-ANCOVA for collapsed strain and strain-separated data, respectively. Effects at the level of the individual strains were assessed as sex-collapsed and for males and females separately. If necessary, a Bonferroni correction for multiple comparisons was included in the ANCOVA. To quantify effect size, Cohen’s *D* was calculated as the daytime mean subtracted from the nighttime mean, divided by the pooled standard deviation *D* = $\frac{\mu_{\text{Night}}-\mu_{\text{Day}}}{\sigma_{\text{p}}}$. The effect sizes were defined as *small D* ≥ 0.2; *medium D* ≥ 0.5; *large D* ≥ 0.8; *very large D* ≥ 1.2 and *huge D* ≥ 2.

(4) Sex effects were calculated based on RM-ANCOVA for collapsed strains as well as separated strains. To quantify the effect size in repeated measures, Cohen’s effect size *f* was calculated from partial Eta squared $\eta_{\text{p}}^{2}$ as *f* = $\sqrt{\frac{\eta^{2}}{1-\eta^{2}}}$ and defined as *large effect* > 0.4, *medium effect* > 0.25, and *small effect* > 0.1.

(5) Between-strain effects were assessed with ANOVA in combination with a Tukey *post hoc* test to identify the strains with homogenous means. The distinction was made based on a *p* = 0.05. Depending on whether the trait underlay a sex-effect (see Table 2), we obtained and illustrated the analysis either for collapsed sexes or males and females separately. Due to the negligible sex effect for feeding the respective figure displays pooled sexes.

(6) Correlations between the traits were assessed by Pearson product-moment correlations. To judge significance, a Bonferroni-corrected *p*-value of *p* = 0.000292 was applied, as there were 171 possible comparisons of 19 traits.

## Results

### Body Weight Is a Sexual Dimorphic Trait and Highly Variable With the Strain Background

**FIGURE 1:**
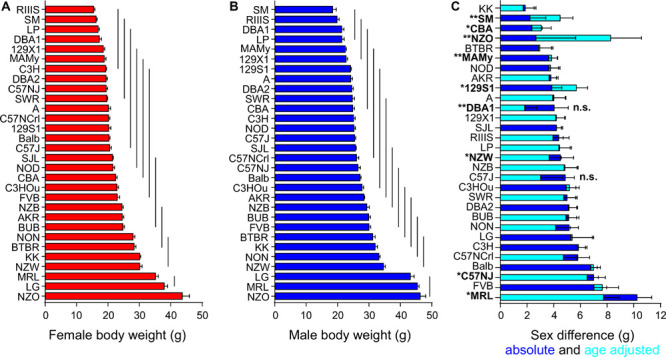
Body weight is a sexual dimorphic trait and varies with the strain background. Body weight distribution among females in red **(A)** and males in blue **(B)** of the 30 inbred strains and quantified sex difference **(C)**. In all strains, males (shown as blue bars) had higher body weight than females. Horizontal bars on columns in **(A,B)** represent standard errors of the mean and in panel **(C)** the standard error of the difference. The vertical lines in **(A,B)** connect groups with homogenous means according to Tukey’s *post hoc* test. The age-adjusted sex difference for all strains is illustrated in cyan. “n.s.” not significant sex difference in body weight, all other strains are significant at a *p*-level < 0.05. Asterisks and bold font indicate a significant age difference, *p < 0.05, **p < 0.001.

Body weight showed a broad distribution across the strains and, as expected, underlay a highly significant strain and sex effect (Figures 1A,B). We calculated an univariate ANCOVA with sex and strain as between-subjects factors and age as covariate. Strain accounted for 90.8% and sex for 52.7% of the variance of body weight (partial Eta squared, see Table 2A). The sex effect on body weight corresponds to a large size effect of *f* = 1.06. The influence of strain and sex on body weight were *F* = 207 and *F* = 681 with a significant interaction of strain and sex (*F* = 2.90, partial Eta squared 12.1%, Table 2A).

**TABLE 2A:** Influence of main factors strain and sex on the respective traits reported as *F*-values and partial Eta squared.

**Trait**	**Body mass**			**Age**			**Strain**			**Sex**			Strain*sex		
	***F***	***p***	**$\eta_{p}^{2}$**	***F***	***p***	**$\eta_{p}^{2}$**	***F***	***p***	**$\eta_{p}^{2}$**	***F***	***p***	**$\eta_{p}^{2}$**	***F***	***p***	**$\eta_{p}^{2}$**
Feeding	2.12	0.15	0.004	2.68	0.10	0.005	12.53	**1.2E-44**	0.382	4.91	**0.027**	0.008	1.17	0.25	0.055
Drinking	0.22	0.64	0.000	0.08	0.78	0.000	8.81	**1.2E-30**	0.304	0.00	0.95	0.000	0.83	0.73	0.039
Fine movements	0.56	0.46	0.001	0.29	0.59	0.000	22.33	**7.1E-77**	0.517	3.76	0.053	0.006	1.98	**0.0018**	0.087
Amb. movements	0.04	0.85	0.000	0.10	0.75	0.000	32.51	**1.2E-103**	0.610	25.86	**4.9E-07**	0.041	2.79	**2.6E-6**	0.118
Rearing	3.64	0.057	0.006	0.00	0.98	0.000	25.54	**3.2E-85**	0.557	42.77	**1.3E-10**	0.068	3.49	**5.3E-9**	0.146
RER	5.81	**0.016**	0.010	0.02	0.90	0.000	7.39	**4.3E-25**	0.263	0.58	0.45	0.001	2.15	**5.4E-4**	0.094
EE	54.54	**5.2E-13**	0.084	0.74	0.39	0.001	13.04	**1.2E-46**	0.388	2.28	0.13	0.004	0.67	0.90	0.032
Cage temperature	4.08	**0.044**	0.007	2.76	0.10	0.005	7.28	**1.1E-24**	0.257	13.24	**2.9E-4**	0.021	1.53	0.038	0.068
Body weight	–	–	–	86.91	**2.1E-19**	0.125	681.00	**2.2E-101**	0.527	207.30	**7.5E-294**	0.908	2.90	**9.8E-7**	0.121

*Body mass and age were registered as covariates in the RM-ANCOVA (for photo- and subsequent scotophase). As for body mass, age was the sole covariate in univariate ANCOVA. “*p*,” *p*-value; “*F*,” *F*-value; “η,” partial Eta squared. Note that the largest effect of age and sex occurred on body weight. The largest sex effect occurred for rearing. EE, average energy expenditure (kcal/h). *P*-values < 0.05 are highlighted in bold.*

The average age of mice investigated in the study was 74.5 ± 11 days. We assessed the influence of age as covariate on all traits and identified, as expected, a significant influence of age on body weight (*F* = 86.91), accounting for 12.5% of the observed variance, but no other trait was sensitive to the differences in age. Due to the limited number of metabolic cages, the high number of strains and the variable breeding performance of each strain, we analyzed mice aged from on average 62 (MAMy) to 92 days (SJL, Table 1). The average body weight and age per strain are displayed in Table 1. The differences in mouse age became significant in 9 strains, including 129S1 (*p* = 0.0022), C57NJ (*p* = 0.036), CBA (*p* = 0.011), DBA1 (*p* = 2.3E-4), MAMy (*p* = 7.4E-5), MRL (*p* = 0.0029), NZO (*p* = 7.8E-5), NZW (*p* = 0.0088) and SMJ (*p* = 3.7E-4).

The sex difference in body weight was highly significant overall and the strain means were for females 23.6 ± 6.9 g and males 28.3 ± 7.1 g. A strain-separated ANCOVA with age as covariate indicated two exceptions that did not display any sex difference: C57J (*p* = 0.079; 3.04 ± 1.65 g) and DBA1 (*p* = 0.054; 1.84 ± 0.90 g). The effect size f indicated a large size effect of sex on body weight for all other strains (*f* > 0.46, *p* < 0.046) and the largest sex effect was observed in Balb (*f* = 3.24), MAMy (*f* = 2.27), SJL (*f* = 2.16), and C3H (*f* = 2.09). Figure 1C quantifies the actual sex difference with correction for age according to ANCOVA.

The lowest mean body weight in males occurred in SM with 18.44 ± 3.44 g and in RIIIS females with 15.43 ± 1.35 g. The NZO was the strain with the highest mean body weight in both males and females (43.66 ± 7.95 g in females and 46.33 ± 5.52 g in males, Table 1 and Figure 1). The strain comparison of mean body weight values resulted in 11 homogenous groups in females and 13 groups in males. The homogenous group with the lowest body weight in males included SM, RIIIS, DBA1, and LP, which also represented the 4 strains with the lowest body weight in females. In both sexes, the group with the highest body weight comprised the three strains LG, MRL, and NZO. Notable, female NZO mice were significantly heavier than MRL and LG females (see Table 3). Figures 1A,B illustrate the distribution of body weight for both sexes across the strains and indicate the homogenous groups. For the further analysis of all other traits, body weight was included as a covariate in the ANCOVA. For most traits its effect seemed small or negligible, except for EE and RER.

### Food and Water Intake Are Strain-Dependent, but Not Sexual Dimorphic Traits

**FIGURE 2:**
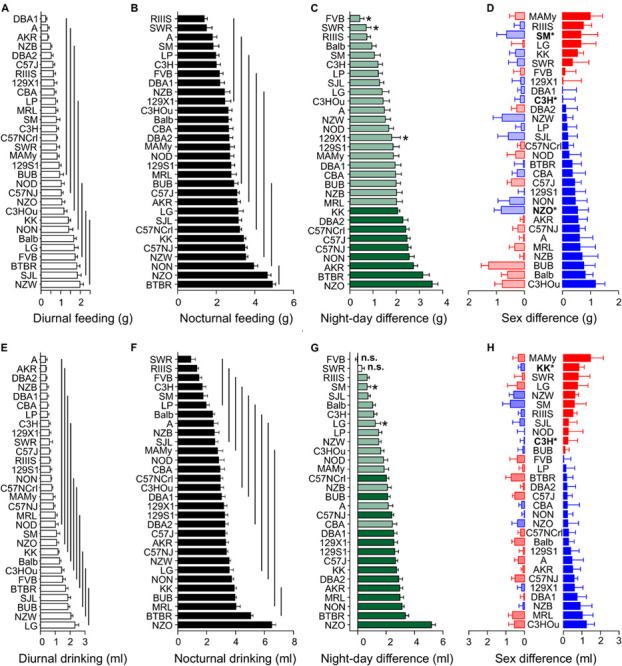
Food and water intake are strain-dependent but not sexually dimorphic. Distribution of diurnal **(A,E)** and nocturnal **(B,F)** feeding **(A,B),** and drinking **(E,F)** behavior among 30 inbred strains. The values are given as mean ± SEM averaged from both sexes. The vertical lines in **(A,B,E,F)** connect groups with homogenous means according to Tukey’s *post hoc* test. **(C,G)** Day–night differences were ranked and Cohen’s *D* calculated. Significant differences at *p* < 0.03 are indicated with asterisk and *p*-levels < 0.001 were not marked; “n.s.” not significant. The effect sizes ranged between *D* = 0.46 (FVB, drinking) and 4.54 (NON, drinking) and are illustrated in light green (*D* = 0.46 and *D* < 2) or in dark green (*D* ≥ 2). **(D,H)** Sex differences were only apparent for food intake in three strains at a *p*-level of *p* < 0.05. The strains are ranked from largest to smallest sex difference for the scotophase, beginning, at the top, with strains, where females (red) consumed more water or food than males, while blue columns illustrate the opposite. To the left, nocturnal values are associated with the respective diurnal values and are displayed in light colors. All values are displayed as means ± standard error of the difference. Asterisks and bold font indicate significant sex differences, *p < 0.05.

Although body weight is sex-dependent, we observed only a small sex difference for food (*F* = 4.91, *p* = 0.03) and no influence of sex on water intake (Table 2). Sex difference explained only 0.8% of the variance in food intake. In contrast, the *F*-values of strain influence were 12.53 and 8.81 for food and water intake, respectively, which accounts for 38.2 and 30.4% of the observed variability (Table 2). As expected, the differences between diurnal and nocturnal food and water intake were large (Figure 2); the differences between the diurnal and nocturnal means were 1.42 (food) and 1.31 (drink) fold larger than the pooled standard deviation (see Table 3) which classifies as very large effect according to Cohen (Sawilowsky, 2009).

Similar to body weight, food and water intake were continuously distributed across the strains and the overall means for diurnal and nocturnal feeding were 1.0 ± 0.78 g and 2.83 ± 1.24 g, and for drinking 1.0 ± 0.83 ml and 3.02 ± 1.55 ml, respectively (Table 3). A strain analysis resulted in distribution in 7 homogenous groups for day and nighttime feeding and 10 and 11 homogenous groups for daytime and nighttime drinking, respectively. The group with the least food intake during the night included RIIIS, SWR, A, SM, LP, C3H, DBA1, NZB, and 129X1 which were all in the group with the least food intake during the day except for FVB. The group with the highest food consumption at night included BTBR, NZO, and NON, which were, except for NON, also at daytime in the group with the highest consumption (Figures 2A,B).

Due to the high correlation between feeding and drinking [*R* = 0.72 (day) and 0.82 (night), see Table 4] the strain distribution was very similar for drinking. In detail, the largest amount of water was consumed at night by NZO, BTBR, MRL, BUB, and KK (NZO: 6.50 ± 1.33 ml; BTBR: 5.05 ± 0.82 ml). There was little correlation between daytime drinking and nighttime feeding values (see below), therefore the group distribution was different during the day and the strains with the highest diurnal water consumption were LG, NZW, BUB, SJL, and BTBR (LG: 2.32 ± 1.25 ml; NZW: 2.05 ± 0.65 ml). SWR, RIIIS, FVB, C3H, SM, and LP consumed the least water at night (SWR: 0.90 ± 1.36 ml; RIIIS: 1.32 ± 0.54 ml) and A and AKR during day (A: 0.35 ± 0.52 ml; AKR: 0.37 ± 0.23 ml). Similar to water consumption, BTBR and NZO consumed most food at night (BTBR: 4.91 ± 0.80 g; NZO: 4.65 ± 0.93 g) and SJL and NZW during the day (SJL: 1.85 ± 0.93 g; NZW: 1.98 ± 0.85 g). In contrast RIIIS and SWR consumed least during the night (RIIIS: 1.38 ± 0.74 g; SWR: 1.50 ± 1.22 g) and A and AKR, including DBA1 consumed the least during the day (A: 0.34 ± 0.35 g; AKR: 0.34 ± 0.25 g; Figure 2).

To visualize the day–night differences in feeding and drinking behavior, we ranked the strains in the order of increasing day–night difference (Figures 2C,G). As for drinking behavior, in almost all strains, with the exception of FVB and SWR, all observed effects were at least medium size effects and associated with *p* < 0.024. The largest day–night differences were observed in the NZO and BTBR strain with 5.26 ± 1.28 ml and 3.35 ± 1.20 ml. NON (3.08 ± 0.68 ml difference) and NZO showed the largest effect size (*D* = 4.54 and 4.10; *p* < 1.5E-12), and the least effect and difference occurred in SMJ and LG (0.66 ± 1.23 and 1.24 ± 1.79 ml difference; *D* = 0.54 and 0.69; *p* < 0.024). In terms of feeding, the largest day–night difference concerned NZO and BTBR (3.59 ± 1.18 and 3.14 ± 1.29 g) and the least FVB and SWR (0.43 ± 0.94 and 0.71 ± 0.88 g). The KK strain showed the largest effect size (2.07 ± 0.50 g difference; *D* = 4.12) and, the FVB strain the smallest effect (0.43 ± 0.94 g difference; *D* = 0.46, *p* = 0.021).

The observation of the negligible sex effect for feeding behavior and the absence of any difference for drinking seemed to contrast the impressive weight differences between males and females. At the individual strain level, the absence of a sex effect was largely confirmed. For feeding only NZO (*p* = 0.001), SM (*p* = 0.04), and C3H (*p* = 0.02) had a marginal sex effect and with respect to drinking KK (*p* = 0.01) and C3H (*p* = 0.02) (Figures 2D,H).

### Physical Activity Traits

Locomotion is linked to complex traits such as general health, exploratory behavior, anxiety and novelty seeking, which is the desire to experience novel stimuli and events, and thus the potential future preference for drugs of abuse (Wahlsten et al., 2006; Wingo et al., 2016). In so far, variability in physical activity are highly relevant for behavioral readouts in psychological and other behavioral tests that aim to quantify these parameters and aim to define environmental and genetic contributing factors. Our measurement setup allowed us to differentiate three types of physical activity: (a) fine motor activity which includes stationary movements like grooming (measured when the same light beam is interrupted more than once); (b) ambulatory activity, which comprises locomotion in the horizontal plane like walking (counted with consecutive interruption of different beams) and, (c) rearing, which is counted when the upper of two light beams is interrupted and this trait includes vertical movements usually considered as exploratory behavior.

#### Fine Motor Activity Is Strain-Dependent but Is Not a Sexual Dimorphic Trait

**FIGURE 3:**
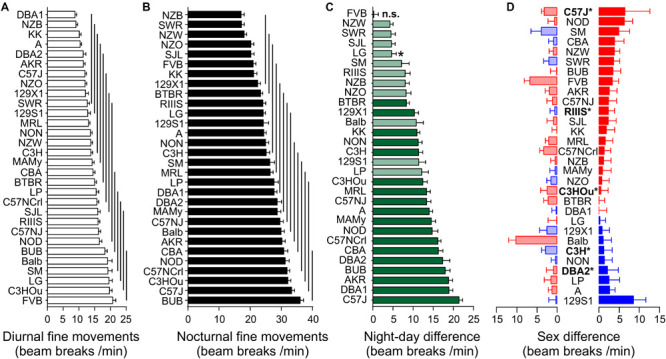
Strain, day–night and sex differences of fine movements. Strain-dependence of diurnal **(A)** and nocturnal **(B)** fine movements. The values are presented as mean ± SEM, averaged from both sexes. The vertical lines connect groups with homogenous means according to Tukey’s *post hoc* test. **(C)** Day–night differences were ranked and Cohen’s *D* calculated. Significant differences at *p* < 0.0016 are indicated with asterisk and *p*-levels < 0.001 were not marked; “n.s.” not significant. The effect sizes ranged from *D* = 0.80 (LG) to *D* = 4.96 (C57J) and are indicated in light green (*D* = 0.8 and *D* < 2) and dark green (*D* ≥ 2). **(D)** Sex differences in fine-motor behavior were apparent in five strains at *p* < 0.05. The strains are ranked from largest to smallest sex difference for the scotophase, beginning, at the top, with strains, where females (red) were more active than males, while blue columns illustrate strains with more active males. To the left, nocturnal values are associated with the respective diurnal values and are displayed in light colors. All values are displayed as means ± standard error of the difference. Asterisks and bold font indicate significant sex differences, *p < 0.05.

Of the three behavioral traits, fine motor activity showed no sex difference, but a strong influence of strain background with *F* = 22.33 (Table 2A). Partial Eta squared indicated that strain background accounted for 51.7% of the variability. The differences between diurnal and nocturnal fine motor activity were highly significant (Figures 3A–C) and classified as very large size effect (*D* = 1.57). The trait values were evenly distributed across the strains and the overall means were 14.41 ± 4.69 during day and 26.05 ± 6.93 during nighttime with female activity levels being one unit higher than males, respectively (see Table 3).

Day- and nighttime fine motor activity in the 30 strains was represented in 13 and 14 homogenous groups, respectively (Figures 3A,B). The group with the highest number of nocturnal fine motor counts comprised the strains BUB, C57J and C57NCrl, C3HOu, NOD, and CBA. C3HOu and BUB were also among the strains with the highest activity during daytime, together with LG, SM, and Balb. FVB showed the highest daytime activity and was the only strain with no day–night difference for this trait (*p* = 0.84). The smallest, but still significant day–night difference were identified in NZW, SWR, SJL, and LG, while the highest differences were measured in C57J, DBA1, and AKR. In all strains with significant day–night difference the observed effect size was large (*D* > 0.80), very large or huge (*p* < 0.0015; Figure 3C). The nocturnal activity levels ranged between 36.06 ± 1.07 (BUB) and 17.11 ± 0.96 counts/min (NZB) and the diurnal extremes were between 20.74 ± 0.97 (FVB) and 8.77 ± 0.51 (DBA1). When we assessed the day–night difference at the sex-separated level, we noticed that not only mice of the FVB strain lacked a day–night difference in activity, but also LG females (*p* = 0.066) and males of the NZW (*p* = 0.076), the SM (*p* = 0.09) and SWR strain (*p* = 0.19).

Regarding the individual strain level, we identified five strains with sex difference (*f* ≥ 0.50). These strains were DBA2 (*p* = 0.044), C3H (*p* = 0.030), C57J (*p* = 0.023), C3HOu (*p* = 0.013), and RIIIS (*p* = 0.0081). In most cases female mice were more active than males (Figure 3D).

#### Ambulatory Activity and Rearing Share Large Strain and Small Sex Effect

**FIGURE 4:**
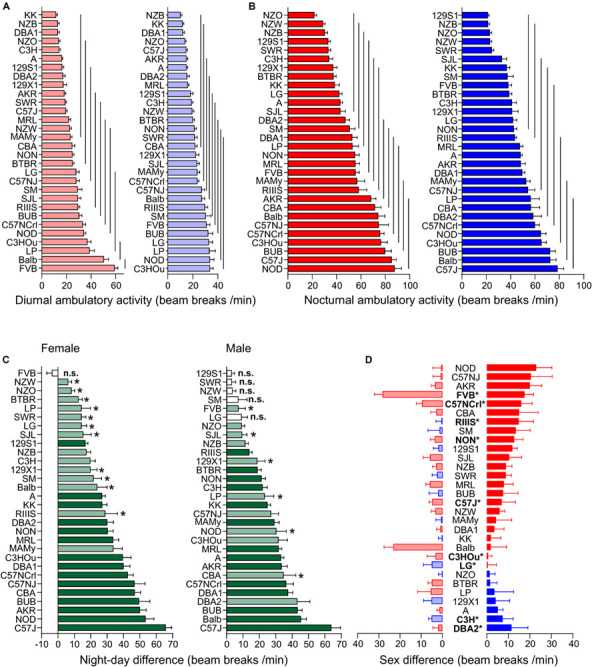
Strain, day–night and sex differences in ambulatory activity. **(A,B)** Strain-dependence of diurnal **(A)** and nocturnal **(B)** ambulatory motor activity. The values are given as mean values averaged from females (left, red) and males (right, blue) with standard errors of the mean. The vertical lines connect groups with homogenous means according to Tukey’s *post hoc* test. **(C)** Day–night differences were ranked and Cohen’s *D* calculated. Differences were indicated with one asterisks at *p* < 0.05; *p*-levels < 0.001 were not marked; “n.s.” not significant. The effect sizes ranged from *D* = 0.48 (FVB males) to *D* = 5.09 (C57J females) and are indicated in light green (*D* = 0.48 and *D* < 2) and dark green (*D* ≥ 2). **(D)** Sex differences were apparent in nine strains at *p* < 0.05. The strains are ranked from largest to smallest sex difference for the scotophase, beginning, at the top, with strains, where females (red) were more active than males, while blue columns illustrate strains with more active males. To the left, nocturnal values are associated with the respective diurnal values and are displayed in light colors. All values are displayed as means ± standard error of the difference. Asterisks and bold font indicate sinificant sex differences, *p < 0.05.

**FIGURE 5:**
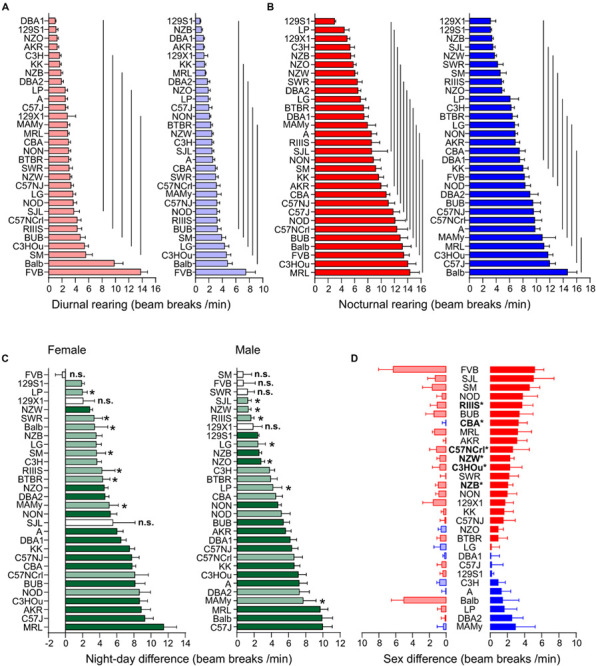
Strain, day–night and sex differences in rearing. **(A,B)** Strain-dependence of diurnal **(A)** and nocturnal **(B)** rearing activity. The values are given as mean values averaged from females (left, red) and males (right, blue), both sexes with standard errors of the mean and the vertical lines connect groups with homogenous means according to Tukey’s *post hoc* test. **(C)** Day–night differences were ranked and Cohen’s *D* calculated. Significant differences at *p* < 0.05 are indicated with asterisk and *p*-levels < 0.001 were not marked; “n.s.” not significant. The effect sizes ranged from *D* = 0.66 (Balb females) to *D* = 5.35 (CBA females) and are indicated in light green (*D* = 0.66 and *D* < 2) and dark green (*D* ≥ 2). **(D)** Sex differences were apparent in six strains at *p* < 0.05. The strains are ranked from largest to smallest sex difference for the scotophase, beginning, at the top, with strains, where females (red) were more active than males, while blue columns illustrate strains with more active males. To the left, nocturnal values are associated with the respective diurnal values and are displayed in light colors. All values are displayed as means ± standard error of the difference. Asterisks and bold font indicate significant sex differences, *p < 0.05.

Ambulatory motor activity and rearing both had very large strain (*F* = 32.51 and *F* = 25.54) and robust sex effects (*F* = 25.86 and *F* = 42.77, see Table 2A). Strain background accounted for 61.0 or 55.7%, respectively, of the variability and sex difference for 4.1 or 6.8%, respectively. For both traits we also observed robust *strain^∗^sex* interactions (see Table 2A). The differences between diurnal and nocturnal activity were highly significant in both traits (Figures 4, 5) and classified as very large size effect (*D* = 1.29 and *D* = 1.26, Table 3). The trait values were continuously distributed across the strains and the overall means were for ambulatory counts 23.53 ± 11.71 during day and 50.07 ± 21.86 during nighttime with the females having on average 3 and 7 counts higher values than the males at day and nighttime, respectively. For rearing the counts were lower with 3.10 ± 2.68 during day and 8.27 ± 4.29 during the night with females having one count higher values than the males in both measurements (see Table 3).

Day and nighttime ambulatory activity in the 30 strains was represented in 10 and 12 homogenous groups in females and 9 and 8 in males, respectively (Figures 4A,B). The distribution appeared rather continuous and the highest number of daytime ambulatory counts occurred in FVB and Balb females and in LP, NOD, and C3HOu males. FVB was again the only strain with no day–night difference for this trait (*p* = 0.51). During nighttime the strains with the highest counts were, similar to fine motor behavior, males and females of C57 strains, BUB, NOD, and Balb. The smallest, but still significant day–night difference occurred in NZW (*D* = 0.64) and SM (*D* = 0.84) and the largest differences were observed in C57J (*D* = 3.92) and A (*D* = 3.91). The nocturnal activity levels ranged in females between 21.65 ± 6.84 (NZO) and 87.43 ± 19.65 counts/min (NOD) and in males between 21.12 ± 3.55 (129S1) and 78.33 ± 18.42 counts/min (C57J). The diurnal extremes were in females between 11.43 ± 4.28 (KK) and 58.96 ± 9.52 counts/min (FVB) and in males between 10.00 ± 4.66 (NZB) and 33.60 ± 9.56 counts/min (C3HOu; Figures 4A,B). When we assessed the day–night difference at the sex-separated level, we noticed that FVB males, but not the females, had a day–night difference (*p* = 0.02, *D* = 0.48). Furthermore, males of 129S1 (*p* = 0.14), LG (*p* = 0.051), NZW (*p* = 0.18), SM (*p* = 0.16), and SWR (*p* = 0.22) lacked the day–night difference in ambulatory activity.

At the individual strain level, we identified six strains with sex difference and these are outlined in Figure 4D. These strains were, in order of decreasing effect size, FVB (*p* = 0.0022), NON (*p* = 0.022), RIIIS (*p* = 0.033), C57NCrl (*p* = 0.020), C3H (*p* = 0.024), DBA2 (*p* = 0.045), C57J (*p* = 0.044), C3HOu (*p* = 0.044), LG (*p* = 0.049). In most cases the females classified with higher activity. As Figure 4D illustrates, the sex effect is mostly attributed to nighttime differences. These specific strains showed large size sex effects (*f* ≥ 0.47).

Diurnal and nocturnal rearing motor behavior in the 30 strains was represented in 7 and 11 homogenous groups in females and 6 and 9 in males, respectively (Figures 5A,B). The highest number of daytime counts occurred likewise in FVB and Balb. FVB appeared again without day–night difference for this trait (*p* = 0.83) and 129X1 were marginally significant, but, when analyzed at the sex-separated level, both sexes lacked a day–night difference. Similar to fine motor and ambulatory behavior, males and females of the C57 strains were among the strains with high numbers of nighttime counts. The smallest, but still significant day–night difference occurred in SJL (*D* = 0.55) and SM (*D* = 0.64) and the largest differences were observed in KK (*D* = 3.19) and DBA1 (*D* = 2.86). The nocturnal rearing activity levels were comparable in both sexes and ranged in females between 2.98 ± 0.61 (129S1) and 14.33 ± 4.91 counts/min (MRL) and in males between 3.14 ± 2.25 (129X1) and 14.65 ± 4.62 counts/min (Balb). The diurnal extremes were in females between 0.99 ± 0.38 (DBA1) and 13.78 ± 3.51 counts/min (FVB) and in males between 0.68 ± 0.36 (129S1) and 7.47 ± 4.89 counts/min (FVB; Figures 5A,B). When we assessed the day–night effect at the sex-separated level, we noticed that not only males and females of FVB lacked a day–night difference in activity, but also males and females of the 129X1 strain (*p* = 0.08 and 0.10) and SJL females (*p* = 0.09) as well as, similar to both other activity counts, males of the SM and SWR strain (*p* = 0.48 and *p* = 0.14; Figure 5C).

We identified six strains with robust sex difference and these are outlined in Figure 5D. These strains were, in order of decreasing effect size, CBA (*p* = 0.0024), NZB (*p* = 0.0068), C57NCrl (*p* = 0.0069), NZW (*p* = 0.011), C3HOu (*p* = 0.024), and RIIIS (*p* = 0.044). In all cases the females classified with higher activity and the sex effect appeared more obvious during nighttime (Figure 5D). These effects were all classified as large size effects (*f* ≥ 0.55).

### RER Varies With Strain and Body Weight, but Is Not Sexual Dimorphic

Respiratory exchange ratio represents the metabolism’s oxidative capacity in combusting carbohydrates or lipids or a mix of both. The value signifies the ratio between the amount of CO_2_ produced and O_2_ used in metabolism. A value of 0.7 is indicative of fatty acids as predominant source of substrate, while values of 1 or larger indicate carbohydrates as the principal fuel source while any value in between represents a corresponding ratio of the two components.

In the present study, the variability in RER underlay a predominant strain effect (*F* = 7.39), a small body mass effect (*F* = 5.81), but no sex effect (see Table 2A). Therefore, strain background accounted for 26.3% and body mass difference for 1.0% of the observed variance, respectively. The differences between diurnal and nocturnal RER were highly significant and classified as very large size effect (*D* = 1.45, Table 3).

**FIGURE 6:**
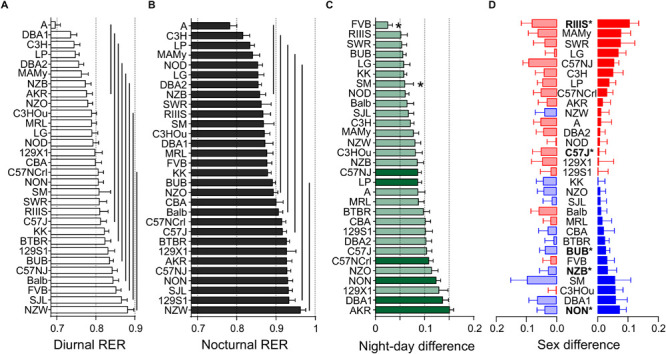
Strain, day–night and sex differences in RER. Distribution of diurnal **(A)** and nocturnal **(B)** RER values. The values are given as mean values averaged from both sexes with standard errors of the mean and the vertical lines connect groups with homogenous means according to Tukey’s *post hoc* test. **(C)** Day–night differences were ranked and Cohen’s *D* calculated. Significant differences at *p* < 0.006 are indicated with asterisk and *p*-levels < 0.001 were not marked. The effect sizes ranged from *D* = 0.54 (FVB) to *D* = 3.71 (AKR) and are indicated in light green (*D* = 0.54 and *D* < 2) and dark green (*D* ≥ 2). **(D)** Sex differences were apparent in five strains at *p* < 0.05. The strains are ranked from largest to smallest sex difference for the scotophase, beginning, at the top, with strains, where females (red) had higher RER than males, while blue columns illustrate the opposite. To the left, nocturnal values are associated with the respective diurnal values and are displayed in light colors. All values are displayed as means ± standard error of the difference. Asterisks and bold font indicate significant sex differences, *p < 0.05.

We obtained a continuous distribution of RER values across the strains with 8 and 6 groups for the day and night values, respectively (Figures 6A,B). A was the strain with the lowest nocturnal (0.70 ± 0.06) and diurnal values (0.78 ± 0.09) and NZW had the highest (0.88 ± 0.08 and (0.96 ± 0.07). To the group with the lowest RER during day and night belonged also C3H, LP, DBA2, MAMy, and NZB. The group with the highest RER comprised 15 strains during day and 14 at night and included, e.g., SJL and 129S1. All strains increased the RER during the night, but when we analyzed the day–night difference at the sex-separated level, FVB females (*p* = 0.98) and males of the SM (*p* = 0.11) and RIIIS (*p* = 0.10) strains showed no difference in substrate choice. The smallest change between diurnal and nocturnal RER was a medium size effect observed in FVB (0.03 ± 0.01, *D* = 0.54, *p* = 0.0052) and the largest occurred in AKR (0.15 ± 0.008, *D* = 3.71, *p* = 3.1E-13; Figure 6C). The average overall values for diurnal and nocturnal RER are given in Table 3. We identified 5 strains with significant sex difference. These strains were, in order of decreasing effect size, RIIIS (*p* = 0.039), C57J (*p* = 0.019), BUB (*p* = 0.033), NON (*p* = 0.035), and NZB (*p* = 0.040). These effects were all classified as large size effects (*f* ≥ 0.49; Figure 6D). BUB, NZB, and NON were the strains where males had a higher RER than females.

### Energy Expenditure

Energy expenditure of laboratory mice is fractioned into the energy required for basal metabolic rate (assessed in resting and fasted animals at thermoneutrality of 29–31°C), thermogenesis (shivering and non-shivering), nutrient digestion (the thermic effect of food) and activity-related energy demands. Our mice were all housed several degrees below thermoneutrality and obtained food *ad libitum*, therefore the total and resting metabolic rates are considered more than two times larger due to the demand for thermogenesis (Even and Blais, 2016). Apart from that, differences in body mass and body composition influence the metabolic rate and contribute to the strain differences.

We interpreted the strain and sex differences in EE after weight adjustment of the EE values with the ANCOVA. The figures therefore display the ANCOVA-predicted values for EE (kcal/h) and the tables show the estimated marginal means.

ANCOVA-adjusted EE showed a very large strain effect (*F* = 13.04) but no significant sex effect (*F* = 2.28, see Table 2A). While age did not influence EE, body weight did largely (*F* = 54.54). Taken together, strain background accounted for 38.8% of the variability, sex difference for 0.4%, and body mass for 8.4%.

**FIGURE 7:**
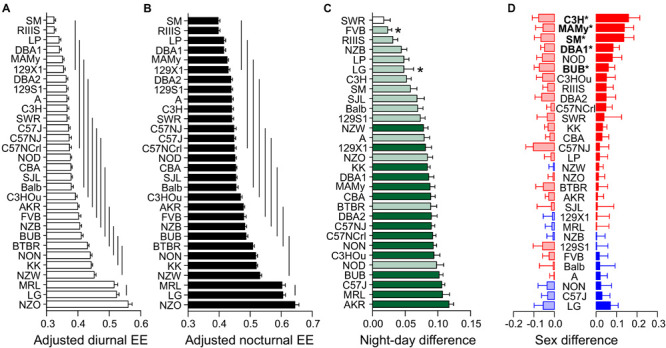
Strain, day–night, and sex differences in energy expenditure. EE was measured as kcal/h and adjusted for body mass and age differences by ANCOVA. **(A,B)** ANCOVA-predicted values of EE illustrate the differences of diurnal **(A)** and nocturnal EE **(B)** between the inbred strains. The values are given as mean values averaged from collapsed sexes. Error bars represent standard errors of the mean and the vertical lines connect groups with homogenous means according to Tukey’s *post hoc* test. **(C)** Day–night differences were ranked and Cohen’s *D* calculated. Significant differences at *p* < 0.004 are indicated with asterisk and *p*-levels < 0.001 were not marked; “n.s.” not significant. The effect sizes ranged from *D* = 0.62 (LG) to *D* = 4.97 (C57J) and are indicated in light green (*D* = 0.62 and *D* < 2) and dark green (*D* ≥ 2). **(D)** Sex differences were apparent in five strains at *p* < 0.02. The strains are ranked from largest to smallest sex difference for the scotophase, beginning, at the top, with strains, where females (red) had higher EE than males, while blue columns illustrate the opposite. To the left, nocturnal values are associated with the respective diurnal values and are displayed in light colors. All values are displayed as means ± standard error of the difference. Asterisks and bold font indicate significant sex differences, *p < 0.05.

The differences between diurnal and nocturnal EE were highly significant and classified as very large size effect (*D* = 1.65, Table 3 and Figures 7A–C). The estimated marginal mean for pooled sexes was 0.397 ± 0.0018 kcal/h at daytime and 0.472 ± 0.0024 kcal/h at nighttime. Females showed, on the strain-separated level, on average slightly higher predicted values than males during both day and nighttime (see Table 3 and Figure 7D).

**FIGURE 8:**
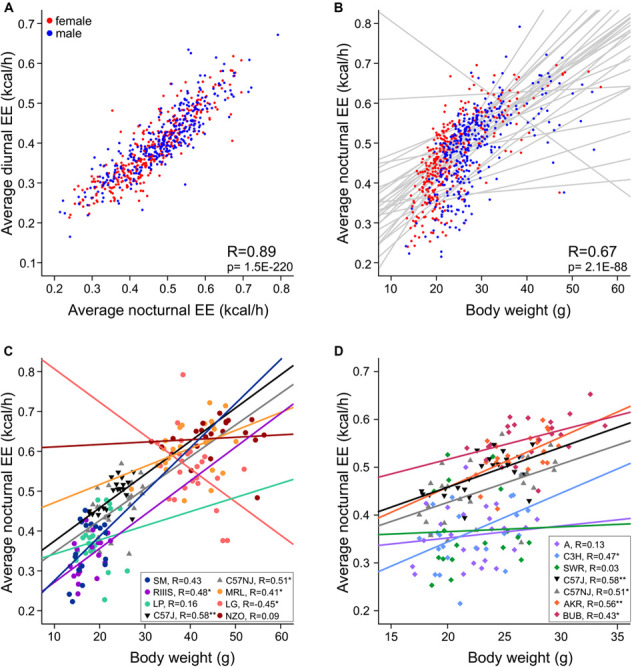
Regression slope visualization of strain-associated effects on EE. **(A)** Nocturnal and diurnal EE are highly correlated. **(B)** Nocturnal EE plotted against body mass with the regression lines for the 30 strains in gray shows striking interaction effects. **(C,D)** Differences in EE plotted for strains with opposite phenotypes for body mass **(C)** or opposite phenotypes for EE but similar body mass **(D)** illustrates non-homogeneity of the regression slopes suggesting that strain-differences in EE are not the same for all body weights. Pearson’s *R* are indicated for the respective strains to describe the magnitude of the respective linear relationship between EE and body mass. The asterisks indicate the level of significance (*p < 0.05, **p < 0.001).

Diurnal and nocturnal adjusted EE was represented in 13 and 10 homogenous groups (Figures 7A,B). EE was highly correlated between day and night (*R* = 0.89, Figure 8A), therefore strain ranks between day and night were rather similar. The highest values occurred at day and nighttime in the strains which also appeared to have the highest body weight which included NZO, LG, and MRL. The highs occurred in NZO and were 0.64 ± 0.062 kcal/h during night and 0.56 ± 0.059 kcal/h during the day. The lowest EE appeared in strains which also shared a low body mass. These strains included SM, LP, DBA1, MAMy, and 129X1. The lows occurred in SM and were 0.40 ± 0.024 kcal/h during night and 0.32 ± 0.023 kcal/h during the day, (Figures 7A,B).

The only strain without day–night difference was SWR (*p* = 0.10). All other strains showed a day–night difference for this trait with at least *p* ≤ 0.002. FVB and LG appeared with a medium size effect (*D* = 0.70 and 0.62) and the largest differences were observed in C57J (*D* = 4.97) and KK (*D* = 4.67). When we assessed the day–night difference at the sex-separated level, we noticed that FVB lacked a day–night difference in females (*p* = 0.11), but also males of RIIIS (*p* = 0.05) and both sexes of SWR (*p* = 0.11 and *p* = 0.83, Figure 7C).

At the individual strain level, only five strains showed a sex difference as shown in Figure 7D. These were SM (*p* = 0.016, *f* = 0.73), MAMy (*p* = 0.019, *f* = 0.68), BUB (*p* = 0.014, *f* = 0.66), C3H (*p* = 0.010, *f* = 0.64), and DBA1 (*p* = 0.011, *f* = 0.64). Remarkably and as visible from Figure 7D, the females appeared in the majority of strains with higher energy expenditure values.

In our study we observed body weights in a large range from 16 to 45 g. Because the metabolic rate does not linearly increase with body weight, the ANCOVA model is *per se* insufficient and becomes probably less accurate in strains with high body weight differences. The magnitude of the relationship between EE and body weight resulted high with *R* = 0.75 (day) and 0.67 (night), but we observed a non-negligible strain^∗^weight interaction (see Table 2B). This led us to analyze the regression slopes across the strains in order to validate the ANCOVA results for groups of strains with significantly different EE. As visible from Figure 8B, the slopes of the 30 strains appear very different and are not parallel which means that the differences in EE which we observed across strains (Figure 7) may not be constant for the full range of body weights. This may be due to inhomogeneity of variances in some of the strain groups. When comparing strains with very large differences in EE such as NZO, MRL and LG and SM, RIIIS and LP, we note that the regression lines cross (Figure 8C). Therefore, e.g., the significant differences in EE observed by ANCOVA between RIIIS, SM and LP with LG are restricted to the body weight differences and not necessarily due to other strain-induced differences. In contrast the differences in EE observed between the C57 strains and RIIIS (Figure 8C) or between BUB or AKR and AJ, C3H and SWR (Figure 8D) are true for the entire range of body weights and likely induced by strain differences independent of body mass because the lines are parallel.

**TABLE 2B:** Strain and body weight interaction are present in activity traits and EE.

**Trait**	**Body mass**			**Sex**			**Strain**			Strain*weight			Sex*weight		
	***F***	***p***	**$\eta_{p}^{2}$**	***F***	***p***	**$\eta_{p}^{2}$**	***F***	***p***	**$\eta_{p}^{2}$**	***F***	***p***	**$\eta_{p}^{2}$**	***F***	***p***	**$\eta_{p}^{2}$**
Drinking	1.73	0.19	0.003	0.61	0.44	0,001	2.43	**5.7E-05**	0,107	1.841	**0.0051**	0.083	0.20	0.66	0.000
Feeding	0.08	0.78	0.000	6.97	0.09	0,012	2.45	**4.5E-05**	0,108	1.922	**0.0028**	0.087	10.32	**0.0014**	0.017
Fine movements	0.07	0.80	0.000	2.73	0.09	0,005	3.27	**3.7E-08**	0,136	2.469	**3.9E-05**	0.106	1.07	0.30	0.002
Amb. movements	0.01	0.91	0.000	16.18	**6.5E-05**	0,026	4.55	**2.4E-13**	0,179	3.020	**3.4E-07**	0.127	6.94	**0.0086**	0.011
Rearing	2.96	0.09	0.005	13.54	**2.6E-4**	0,022	4.65	**9.3E-14**	0,186	3.309	**2.6E-08**	0.140	3.53	0.06	0.006
RER	13.60	**2.5E-4**	0.022	0.01	0.93	0.000	1.84	**0.0052**	0.081	1.90	**0.0033**	0.084	0.61	0.43	0.001
EE	92.44	**1.9E-20**	0.13	7.90	**0.0051**	0.013	2.67	**7.6E-06**	0.115	2.41	**6.7E-05**	0.105	3.04	0.08	0.005
Cage temperature	0.37	0.54	0.001	1.98	0.16	0.003	2.76	**3.3E-06**	0.116	2.53	**2.3E-05**	0.108	5.00	**0.026**	0.008

**Sex and weight were registered as covariates in the RM-ANCOVA. “p,” p-value; “F,” F-value; “η,” partial Eta squared.* P-values < 0.05 are highlighted in bold.*

### Cage Temperature

**FIGURE 9:**
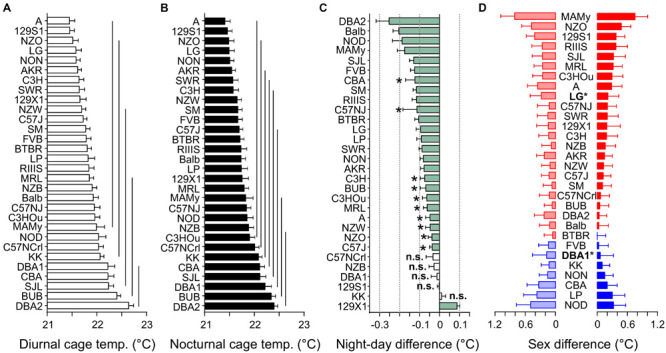
Strain, day–night, and sex differences in cage temperature. Distribution of diurnal **(A)** and nocturnal **(B)** cage temperature values (°C). The values are given as mean values averaged from both sexes with standard errors of the mean and the vertical lines connect groups with homogenous means according to Tukey’s *post hoc* test. **(C)** Day–night differences were ranked and Cohen’s *D* calculated. Significant differences at *p* < 0.05 are indicated with asterisk and *p*-levels < 0.001 were not marked; “n.s.” not significant. The effect sizes ranged from *D* = 0.33 (C57NJ) to *D* = 1.65 (129X1 and SM). **(D)** Sex differences were apparent in two strains at *p* < 0.04. The strains are ranked from largest to smallest sex difference for the scotophase, beginning, at the top, with strains, where females (red) had higher cage temperatures than males, while blue columns illustrate the opposite. To the left, nocturnal values are associated with the respective diurnal values and are displayed in light colors. All values are displayed as means ± standard error of the difference. Asterisks and bold font indicate significant sex differences, *p < 0.05.

**FIGURE 10:**
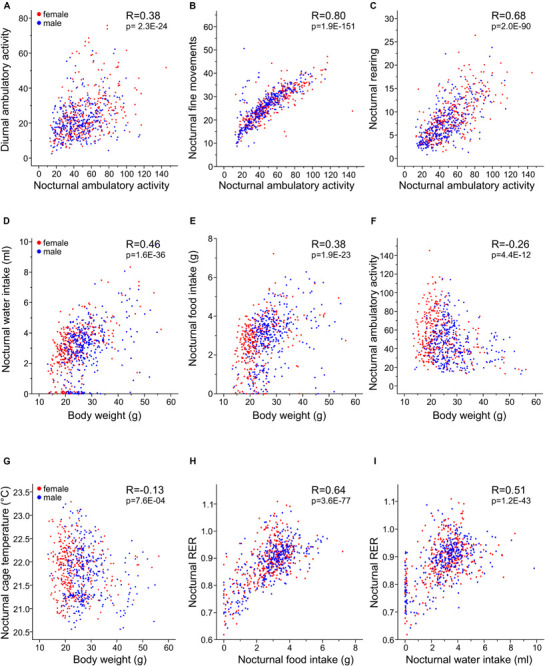
Pearson correlation matrix for selected traits. Illustrations of the relationships between day and nighttime activity parameters **(A)** and cross-correlation of different nocturnal activity traits, all measured as number of light beam breaks per minute **(B,C)**. Body mass correlation with nocturnal water **(D)** and food **(E)** intake, nocturnal ambulatory activity **(F)** and cage temperature **(G)**. Each point represents data from one mouse during one scotophase and its body weight. **(H,I)** The relationships between nocturnal food intake and water intake with RER. Female mice are shown in red and male mice in blue. The correlations of all other parameters are shown in Table 4, the *p*-value was corrected to *p* < 0.000292.

Cage temperature shares a good correlation with body temperature, as previously identified in rats (Urbach et al., 2014), however, such a validation is lacking for mice. Like, RER, cage temperature underlay a strain effect (*F* = 7.28) and a small sex effect (*F* = 13.24, see Table 2A and Figure 9). Strain background accounted for 25.7% of the variance and sex difference for 2.1%. Furthermore there was a small, but significant influence of body mass (*F* = 4.08) which accounted for 0.7% and it was represented in a correlation of day or night cage temperature with body weight of *R* = −0.13 (Figure 10). The differences between diurnal and nocturnal cage temperature were significant, but in contrast to all other traits underlay a medium size effect (*D* = 0.54, Table 3). The temperatures in the cage were consistently warmer during the light cycle, on average by 0.08°C which is in contrast to our previous study in rats (Urbach et al., 2014). 129X1 were the only strain where the opposite was the case, an effect which may have to do with low numbers in this strain (Table 1). The day and nighttime cage temperature distributed in 5 and 8 homogenous groups, respectively. A was the strain with the lowest value during both night (21.40 ± 0.52°C) and day (21.45 ± 0.52°C) and DBA2 with the highest values (22.38 ± 0.35 and 22.22 ± 0.66°C; Figures 9A,B). There were 5 strains without a day–night difference, and these were 129S1 (*p* = 0.88), C57NCrl (*p* = 0.22), DBA1 (*p* = 0.73), KK (*p* = 0.59), and NZB (*p* = 0.15). The largest difference occurred in DBA2 (0.25 ± 0.06, *D* = 0.81) and the smallest, still significant difference was observed in C57J (0.04 ± 0.13, *D* = 0.55, Figure 9C). The observed small sex effect was based on significant differences between only two strains, DBA1 (*p* = 0.033, *f* = 0.53) and LG (*p* = 0.036, *f* = 0.50), but, as visible from Figure 9D, females of most strains had the higher cage temperatures.

**TABLE 3:** Influence of day–night variation and sex on the behavioral traits.

**Trait**	**Sex-collapsed data**					**Sex-separated data**						**Sex effect**	
	**Time**	**Population means**		**Day–night effect**		**Sex**	**Time**	**Population means**		**Day–night effect**			
		**Mean** ± **SD**	***n***	***p***	***D***			**Mean** ± **SD**	***n***	***p***	***D***	***p*-value**	***f***
**Feeding** (g)	Day	1.00.78	649	3.5E-188	1.42	Female	Day	1.020.76	329	1.5E-87	1.37	0.027	0.09
							Night	2.731.18					
	Night	2.831.24				Male	Day	1.000.80	320	2.0E-101	1.49		
							Night	2.931.30					
**Drinking** (ml)	Day	1.00.83	648	9.9E-196	1.31	Female	Day	1.040.84	325	1.8E-93	1.30	0.95	–
							Night	2.991.52					
	Night	3.021.55				Male	Day	0.930.81	323	9.1E-104	1.33		
							Night	3.051.58					
**Fine**	Day	14.414.69	666	5.4E-239	1.57	Female	Day	14.874.97	333	1.2E-108	1.61	0.053	–
**movements**							Night	26.616.88					
(subsequent	Night	26.056.93				Male	Day	13.964.36	333	3.4E-97	1.53		
beam breaks)							Night	25.486.95					
**Ambulatory**	Day	23.5311.71	665	3.9E-205	1.29	Female	Day	25.0812.95	333	1.2E-108	1.35	4.9E-7	0.21
**movements**							Night	53.5622.96					
(consecutive	Night	50.0721.86				Male	Day	21.9710.11	332	3.4E-97	1.25		
beam breaks)							Night	46.5720.13					
**Rearing**	Day	3.102.68	652	2.8E-172	1.26	Female	Day	3.493.12	327	2.6E-85	1.29	1.3E-10	0.27
(*Z*-axis beam							Night	8.994.51					
breaks)	Night	8.274.29				Male	Day	2.702.08	325	1.1E-89	1.25		
							Night	7.543.94					
**RER** (V CO_2_/	Day	0.800.08	663	4.1E-180	1.45	Female	Day	0.800.08	334	1.7E-87	1.42	0.45	–
V O_2_)							Night	0.890.08					
	Night	0.880.08				Male	Day	0.790.08	329	3.0E-94	1.48		
							Night	0.880.08					
**Energy**	Day	0.400.02	658	3.5E-209	1.65	Female	Day	0.400.003	332	5.1E-100	1.65	0.13	–
**expenditure**							Night	0.470.004					
(kcal/h)*	Night	0.470.02				Male	Day	0.390.003	326	2.3E-110	1.66		
							Night	0.470.004					
**Cage**	Day	21.870.56	671	8.2E-43	0.54	Female	Day	21.920.53	337	2.6E-20	0.49	0.00029	0.15
**temperature**							Night	21.850.51					
(°C)	Night	21.790.53				Male	Day	21.820.58	334	2.9E-24	0.59		
							Nnight	21.730.55					
**Body weight** (g)						Female		23.636.94	337			2.2E-101	1.06
						Male		28.257.06	334				

Day–night variation on the behavioral traits are given as diurnal and nocturnal population means for pooled sexes (left columns) and separate sexes (middle columns) across 30 strains. The comparisons follow RM-ANCOVA (as in Table 2). The effect sizes for repeated measures are reported as Cohen’s D [very small D ≥ 0.01; small D ≥ 0.2; medium D ≥ 0.5; large D ≥ 0.8; very large D ≥ 1.2 (Sawilowsky, 2009)] and for sex as f as calculated from the respective partial Eta squared (large effects > 0.4, medium effects > 0.25, and small effects > 0.1). “n,” sample size; “p,” p-value; “D,” effect size; outlier analysis was performed for each strain and sex. Body weight was analyzed using univariate ANOVA. *EE was modeled by ANCOVA with bodyweight and age as covariates and the estimated marginal means are displayed.

### Pearson Correlations Reveal Strong Relationships Between Food Consumption, RER, and Energy Expenditure

For all traits, we identified moderate to high correlations between nocturnal and diurnal measurements (Table 4). For the day–night correlation of the three types of activity, the *R*-values were moderate and between 0.24 and 0.38 (Figure 10A). Diurnal and nocturnal RER were correlated with *R* = 0.73 and diurnal and nocturnal EE with 0.89 (Figure 8A). Cage temperature, feeding and drinking had the highest *R*-values for day- and nighttime data of *R* = 0.96. Therefore, we delineate that in general, activity-related experiments conducted at night or daytime should be correlated, although the results will differ quantitatively.

**TABLE 4:** Correlations among the traits based on collapsed-strain and collapsed-sex data.

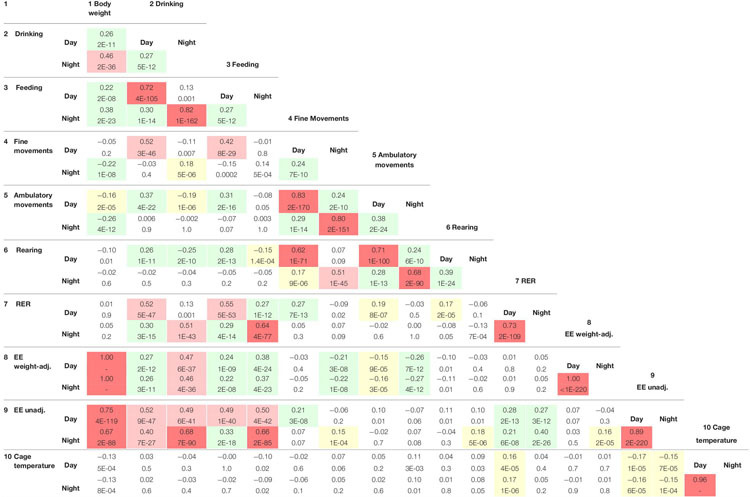

**The sample size per correlation is n = 647–671. The table provides the correlation coefficient and the respective p-value. The significance level was Bonferroni-corrected to p < 0.000292. Color code: white: no correlation, p > 0.000292; yellow: correlations with R ≤ 0.20 and 1E-07 < p < 0.000292; green: correlation with 0.20 < R < 0.40 and 1E-26 < p < 1E-07; light red: correlations with 0.40 < R ≤ 0.60 and 1E-60 < p < 1E-26; dark red correlations with R > 0.6.**

The equipment of the cages with light beams in the horizontal and vertical plane and the analysis with respect to timing of beam breaks allows separation of the activity parameters in three subtypes. Nevertheless, the correlation analysis, showed a high correlation among them, with fine movements and ambulatory activity having the highest correlation of at least *R* = 0.80. In contrast, rearing was higher correlated with ambulatory movements (at least *R* = 0.68) than with fine movements (at least *R* = 0.51, Figures 10B,C and Table 4).

As expected, both drinking and feeding had high correlations with body weight (*R* = 0.46 and *R* = 0.38; Figures 10D,E). In addition, body weight was negatively correlated with physical activity (*R* = −0.26 and *R* = −0.22 for ambulatory and fine movements, respectively), except for rearing which was not related with body weight. In the figure it is clearly discernible that the lightweight females are more active and that heavier bodies are less prone to be physically active with probably reduced contribution to the total energy expenditure (Van Klinken et al., 2012) (Figure 10F). Last but not least and as expected, body mass had a moderate negative correlation with cage temperature (Figure 10G and Table 4).

Respiratory exchange ratio was not correlated with body weight (Table 4), but interestingly, RER had a high correlation with both diurnal and nocturnal food and water intake (at least *R* = 0.55 and 0.51), thus, reasonably, with increasing chow consumption, RER increased and the predominant source of substrate shifted from fatty acids to carbohydrates (Table 4 and Figures 10H,I). In contrast higher levels of activity were not correlated with increased RER. We identified a remarkable correlation between food intake or drinking and ambulatory activity, rearing and fine movements, such as grooming, which was restricted to the day, i.e., during the day higher levels of activity were related with larger amounts of consumed food and more drinking or vice versa (Table 4).

We observed a high positive correlation between the energy expenditure with increasing food and water consumption (nocturnal *R* = 0.38 and 0.47; Table 4). This observation provides the link to the thermic effect of food and to diet-induced adaptive thermogenesis. Therefore, an increase in white adipose tissue leads to an increase in functional active brown adipose tissue, which lowers the energetic efficiency of feeding. The increase in energy-expenditure that results from diet-induced adaptive thermogenesis is mediated via a recently discovered hypothalamic NPY-dependent circuitry, which signals independent of the cold-induced BAT thermogenesis activation (Zhang L. et al., 2018).

## Discussion

Our survey in 30 inbred mouse strains provides a large and comprehensive dataset including in-cage activity parameters, feeding, drinking, RER and measures of EE at normal housing conditions. As expected, for all traits, we found an enormous variability between strains, and, for particular traits, such as body weight, rearing and ambulatory activity also a large difference between sexes. The strain- and sex-separated statistical analysis then identified the particular strains with diurnal or nocturnal behavior and those strains with sex difference. Furthermore, the correlation analysis, based on more than 650 individual data points per trait, delivered a few remarkable insight into associations between specific traits.

The RM-ANCOVA summarized in Table 2, identified that most of the variation in the behavioral traits were due to strain differences, thus explained by genetic effects. Nevertheless, inclusion of body mass as additional covariate attributed some of these effects to strain-body mass interactions. Sex effects became apparent, specifically with regard to body weight, and activity traits (ambulatory and rearing locomotor behavior). One contributor to the variance in body weight was identified to be age, but also litter size contributes (and was not regarded here) because mice from litters with fewer sibling are larger (Cowley et al., 1989). The RM-ANCOVA using weight as cofactor recognized that a large part of the variance observed for EE is due to weight differences, unfortunately we cannot provide a more accurate adjustment of EE, because we have not acquired data on lean body mass to allow for a more advanced analysis (Fernandez-Verdejo et al., 2019). A previous study on body composition in inbred strains showed that heavier mice have more body fat. Fat is poorly metabolically active and contributes less to the energy expenditure than lean body mass (Reed et al., 2007; Tschop et al., 2011; Fernandez-Verdejo et al., 2019). The influence of differences in body composition on EE are therefore probably most pronounced in the strains which are heaviest and have the largest amount of body fat, which include KK, MRL, LG, NON, and NZO [in these strains percent body fat ranges between 25–35%, (Reed et al., 2007)]. These strains may therefore have lower EE than predicted with the RM-ANCOVA here (Figure 7).

We did not generally identify sex as a large contributor to EE (Table 2). Nevertheless, sex differences in EE are the subject of intensive research and recently a study based on a novel 5-HT_2C_R^CRE^ mouse line identified a specific population of pro-opiomelanocortin hypothalamic neurons expressing 5-hydroxytryptamine 2c receptors to drive a large sex difference in physical activity, energy expenditure and the development of obesity (Burke et al., 2016).

**FIGURE 11:**
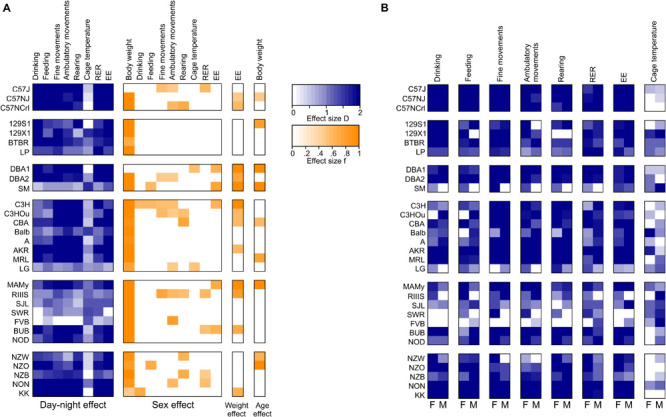
Heat map summarizing for all traits and strains, sex effects and day-night differences for pooled and separate sexes. **(A)** Day–night-differences are given as Cohen’s *D*, sex effects, weight effect on EE and age effect on body weight are given as f and calculated from partial Eta squared $\eta_{\text{p}}^{2}$; **(B)** day–night- differences as Cohen’s *D* reported for separate sexes. White boxes represent insignificant differences.

The 30 inbred strains here included the most common strains available from the Jackson Labs, and as far as the C57BL/6NJ strain is concerned also the breed provided by Charles River laboratories termed the C57BL/6NCrl. The 30 strains belonged to 6 genetically related families (Petkov et al., 2004) and in order to align the differences between strains and sexes, we used the effect sizes and summarized sex, day–night difference, weight and age differences in a heat map (Figure 11) based on the effect size calculated as *D* or from partial Eta squared similar in method to a previous publication (Timotius et al., 2019).

The *C57-related strains* exhibited a quite comparable behavior and belonged in virtually all traits to the same homogenous group. Some small differences appeared for fine movements where the C57J exhibited a sex difference and had markedly lower activity levels during daytime than the C57NJ and C57NCrl. Concerning ambulatory movements, the C57 strains belonged to adjacent homogenous groups, but all three C57 strains showed a robust sex difference with females being more active. In all activity traits, the C57 strains were strongly nocturnal, exhibiting the largest day–night difference of all strains. Thus for, e.g., the measurements of voluntary behavioral traits, such as treadmill exercising, both findings seem of particular importance (Gibb et al., 2016). An important difference between the C57J and C57NJ strains concerns the glucoregulatory response and the control of glucose homeostasis. In this respect the C57J strain is highly susceptible to develop a diabetes mellitus type 2 phenotype with obesity and hyperglycemia in response to a high fat diet. Furthermore, C57J mice develop glucose intolerance on a regular chow diet, a phenotype which is attributed to a loss of function mutation in the nicotinamide nucleotide transhydrogenase gene. While these differences develop stimulus-induced, they don’t seem to overtly affect EE and preferred substrate combustion (RER) in C57 strains (Fergusson et al., 2014).

Other frequently used strains in laboratory research are strains derived from the 129 lineages of *Castle’s strains*. The availability of multiple stem cell lines derived from 129 strains facilitated their use for most null mutant and transgenic overexpression lines as background strain (Petkov et al., 2004). Related with this lineage are also the BTBR and LP strain. Remarkably the BTBR strain, which stand out by absence of the corpus callosum and an autism spectrum disorder–like phenotype (Wahlsten et al., 2003; Meyza and Blanchard, 2017), were at the extreme of the food and water consumption and markedly different from the three other strains. BTBR were recently identified to have a taste receptor mutation in the inositol triphosphate receptor 3 gene which makes them indifferent to sweet and other tastes and it was concluded to also influence their macronutrient choice due to impairment of the detection of nutrients in the diet (Tordoff et al., 2012; Tordoff and Ellis, 2013). In a survey of voluntary calcium intake, the BTBR were also among the strains with the highest consumption of calcium solutions relative to water, in contrast they were the strain that resisted alcohol consumption and always preferred water to alcohol or to sweetened alcohol (Tordoff et al., 2007; Yoneyama et al., 2008). With respect to the activity traits, the BTBR strain behaved comparable to the 129 strains and they were in homogenous groups concerning fine motor behavior and rearing at both day and nighttime. Concerning ambulatory activity, the LP strain differed from the other three and was markedly more active, especially during the light cycle. The 129 strain appeared previously in an analysis of avoidance conditioning as one of the strains with the slowest reaction time and the least amount of correct avoidances together with Balb (Royce, 1972). Therefore, it seemed not surprising that 129S1 and 129X1 had the least amount of rearing counts during both day and night and were also at the low end of the scale for ambulatory behavior. In addition, we observed a sex difference where males of the 129S1 strain lacked a day–night difference for ambulatory behavior and female mice proved more active in general. A recent study of anxiety assessment confirmed a hypoactive phenotype in 129S1 with low locomotor, rearing and exploratory activity (Kulesskaya and Voikar, 2014) and another study corroborated a lack of habituation of anxiety-like behavior in several 129 substrains and attributed a general vulnerability in coping with environmental changes to the 129 genetic background (Boleij et al., 2012). Altogether these findings make 129S1 a difficult strain for behavioral tasks that rely on voluntary behavior, motivation, exploration of novel environments and probably locomotor activity in general.

We included three strains from the *CC Little’s DBA and related strain lineage* which were DBA1, DBA2, and SM. DBA1 and DBA2 originate from the same lab and differ by only 5.6% at the single nucleotide polymorphism (SNP) level which seem to be the cause of a number of metabolic and lipid phenotypes, such as differences in the triglyceride and HDL plasma levels (Stylianou et al., 2008). In our study the strains of this lineage did not differ in body weight, food and water intake, RER and EE. However, SM differed in its diurnal activity behavior from DBA1 and DBA2 in all three locomotor traits, while for nocturnal locomotor activity the strains shared homogenous groups. Most remarkably, in these locomotor traits, the males of SM showed a lack of day–night difference in all traits, fine motor, ambulatory and rearing activity, while the females were clearly nocturnal. A previous study compared activity rhythms between males of the SM and A strain (females were not included in the study) and found that, while A started activity almost at the time of lights-off, SM became active at 3 h before the lights-off and with respect to daily activity counts, the SM were more active than A. This difference, although based on measures of free-running and wheel-running activity, matches our finding for daily ambulatory activity, where males of the A strain showed much less activity than SM (Suzuki et al., 2000).

A are an albino strain of the *Bagg albino lineage*. From this family we also measured the agouti-colored C3H, C3HOu, and CBA and the albino Balb, AKR, MRL, and LG strains. Very remarkable is that mice of the A strain, which are classified as obesity-resistant strain when on a high-fat diet, have the lowest value for RER both during day and night and belong to the group with lowest amount of chow consumption during day and night of all measured strains. Seemingly they rely on lipid oxidation during the day with some additional combustion of carbohydrates during the night when they increase feeding. In fact, when weaned in thermoneutrality, A mice, in contrast to C57J, become hypothermic on 4°C cold exposure on a low fat diet and are rescued when they have access to a high fat diet. In A mice this causes leptinemia and induces fatty acid oxidation in muscle and brown fat as part of non-shivering thermogenesis (Kus et al., 2008). In our study, A was among the strains with relatively low EE and when directly compared to obesity-prone C57J, the EE was not significantly lower during day and night (compare Figure 8D, A, and C57J were also indifferent in body weight in our study). This finding seems in accordance with previous findings (Bardova et al., 2016). In part the low energy expenditure may account for the lower nocturnal activity levels observed in A. Bardova et al. (2016) also found that, after fasting, A were capable to switch to glucose oxidation faster and more extensively in comparison to C57J and they suppose that the higher levels of leptin observed in A may contribute to the higher metabolic flexibility of A mice and they suggest that leptin is also involved in increasing metabolic preference toward triacylglycerol hydrolysis and fatty acid oxidation (Harris, 2014).

The Balb strain is a frequently used mouse model for behavioral studies. While they share the phenotype of slow-conditioning with 129S1 (Royce, 1972), their locomotor behavior in the present study were rather opposite and Balb belonged to the group of mice with high levels of fine motor movements, ambulatory and rearing activity. With respect to fine and ambulatory activity, the females had lower values than the males. Previously Balb were described to have lower sociability as for example C57J (Sankoorikal et al., 2006) and were recognized for highly aggressive inter-male behavior (Dow et al., 2011). The latter trait is similar to NZB and opposite to A and intercross studies were successfully conducted to investigate genomic loci with influence on aggressive behavior (Dow et al., 2011).

The CBA and C3H strains are agouti-colored. C3H substrains resulted from crosses of Balb and DBA. C3H and C3HOu are characterized as genetically very similar, but differ for example in a mutation in the toll-like receptor 4 gene which make C3H endotoxin resistant (Watson et al., 1977). Here, unexpectedly, and unlike to the C57 substrains, there were remarkable differences in C3H substrains. These included nocturnal drinking, fine motor behavior, ambulatory activity, rearing in females and in males during the night, but not EE or body weight. In the named traits, the C3HOu had higher values. Both substrains and CBA share a homozygous mutation in the retinal degeneration 1 mutation *Pde6b*^rd1^ which makes them blind at the age of weaning (Foster et al., 1991; Chang et al., 2002) and is believed to affect learning and memory in these strains (Clapcote et al., 2005). This mutation affects only classical photoreceptor-based vision and not light sensing as it is required for sustaining a light dark cycle, because we found the three strains to be pronounced nocturnal strains in both males and females. The three strains are known to be melatonin-proficient strains. In our analysis, which is based on averaging data from one photo- and one scotophase, they also did not differ in nocturnal and diurnal average values from the melatonin-deficient C57 strains. Nevertheless, this does not rule out that differences between the strains are present at periods that don’t become evident from 12 h averaging (Stehle et al., 2002; Zhang Z. et al., 2018).

The MRL strain is a remarkable strain due to its regenerative abilities that lead for example to scarless healing of punch holes in the ear, replacement of injured heart muscle with normal tissue architecture and enhanced healing responses after spinal cord hemisections that lead to fast and complete recovery of motor function (Thuret et al., 2012; Podolak-Popinigis et al., 2015). Here, they were part of the group with the highest body weight in both males and females which is likely due to their higher fat mass in contrast to other strains, such as SJL (Srivastava et al., 2006). We found them among the strains with the highest EE. In the past they served as intercross with SJL mice to research QTLs for total body fat mass and obesity (Srivastava et al., 2006), because they have opposite phenotypes in serum levels of cholesterol, HDL, TG, and body fat mass. Like MRL, AKR, and LG are heavyweight strains in this lineage. LG were bred and selected for large growth and, as intercross with SM, are used as model to study complex polygenic traits such as body size, skeletal morphology, obesity and response to dietary fat intake (Ehrich et al., 2003).

In a previous study on body composition in 40 inbred strains conducted with Dual-energy X-ray absorptiometry in carcasses, AKR, as well as several strains from the *Japanese and New Zealand lineage*, which are also included here, such as NZW, NZO, NZB, NON, and KK, appeared all among the heavyweight strains similar to one previous study (Reed et al., 2007). Similar to intercrosses of LG × SM and A × C57, the AKR x SWR intercross is selected for phenotype dissimilarity in diet-induced obesity (West et al., 1994; Ehrich et al., 2003; Collins et al., 2004). Nevertheless, these strains are not the heaviest laboratory mouse strains, because previous selective breeding studies (Bunger et al., 2001) produced strains with much heavier body weight than reported for the strains used here or in previous studies (Reed et al., 2007). Several strains of the Japanese and New Zealand mice are used in studies of obesity. KK mice are a polygenic obese mouse model for diabetes mellitus type 2 due to their inherited glucose intolerance and insulin resistance. They develop DMT2 in response to high fat diet and usually during aging (Ikeda, 1994; Herberg and Coleman, 1977; Berndt et al., 2014). Similarly, NZO develop an early onset DMT2 due to obesity and are used as model for peripheral neuropathy and treatment of diabetic neuropathic pain (Zhang et al., 2013). In our study, NZO were the heaviest and among the strains with the largest water consumption, the latter one potentially being a symptom of polydipsia in DMT2. Obesity in this strain is extreme and fat depots exceed 40% of total body weight at 6 months of age. This results from a combination of a moderately increased food intake (hyperphagia), reduced thermogenesis resulting in a reduced body temperature by 1.5°C, reduced EE and reduced voluntary running wheel activity (Jurgens et al., 2006). In our study, NZO were the heaviest strain and had the largest EE. This previous study also compared the energy expenditure of NZO with the closely related lean NZB strain and found that the heavier NZO have higher EE, but they also noted that NZB consume 2.3 g more food (very similar to the 2.2 g in our hands) which makes a comparison difficult. They hypothesized that the total EE in NZO may be lower than in lean NZB due to the lower body temperature and the lower lean mass (Jurgens et al., 2006). In our hands, alongside with KK, NZB, and NZW the NZO were also the least active strains during both night and day. NZB were used as intercross with SM due to their diverging phenotypes in a large number of metabolic phenotypes, body composition and size (Stylianou et al., 2006). This intercross led to the identification of QTLs affecting for example HDL cholesterol and atherosclerosis (Korstanje et al., 2004a, b). Here, NZB among KK, NZW, and NZO appeared to be in the group with the least fine motor and ambulatory movements during the night (Lightfoot et al., 2004). When compared for nocturnal EE, NZO, NON, KK, and NZW were among the strains with the highest values and all significantly higher than NZB.

From the *Swiss lineage*, we measured the albino strains MAMy, RIIIS, SJL, SWR, FVB, BUB, and NOD. In this lineage, most strikingly, several strains had a weak or absent day–night difference in some of the traits and appeared similar to SM. FVB had no day–night difference in all three activity traits. SWR, SJL, and RIIIS had a relatively weak day–night difference in ambulatory and rearing locomotion. In SWR, similar to SM, this was based on a lack of day–night difference in males and, in addition, SWR had no difference in nocturnal and diurnal EE. Similar to C3H and CBA, SJL and SWR carry the *Pde6b*^rd1^ mutation leading to rod receptor dysfunction while RIIIS carry the *ldis1* mutation resulting in cataract formation, but, although blind to visual images, they are capable of maintaining a regular circadian rhythm due to intact photosensing retinal ganglion cells (Pugh et al., 2004).

In a previous study, AKR and SWR were compared in their activity and energy metabolism because in contrast to SWR, AKR are susceptible to diet-induced obesity. SWR were more active (in an open field test), due to increased activity levels during the photophase, but the strains did not differ in the scotophase. SWR dissipate excess energy on a high fat diet by higher activity levels and thermoregulatory behavior as well as subsequent reduction of food intake (Hesse et al., 2010). In our study, SWR had similar activity levels during the photophase as compared to AKR, therefore the in-cage activity readout differs in these strains from the activity in an open field test. Nevertheless, when we correlated the open field *Pletcher1* dataset from Jackson Lab’s mouse phenome database with our total diurnal ambulatory activity, we found, based on male mice of 22 strains which overlapped in our studies, a significant correlation with total distance traveled in the open field (*R* = 0.56, *p* = 0.006) and a significant negative correlation of time spent in the center of the open field (*R* = −0.49; *p* = 0.017). The discrepancy may be due to the finding that in the dataset from *Pletcher1*, AKR, and SWR are found relatively close together and, that we only analyzed average values during photo- and scotophases which may blur stronger differences among the strains.

As far as FVB are concerned, disruption of the usual light-dark cycle has been described and, in addition to the *rd* mutation, a defect in the photosensing cells in the retina is suspected (Foster et al., 1991). Therefore this strain failed in test such as the Morris water maze where spatial learning is required, but also in other tests like fear conditioning and they show altered social behavior with increased aggression (Bolivar et al., 2001; Pugh et al., 2004).

## Conclusion

This survey demonstrated that naturally occurring genetic variations modulate various innate activity behaviors, substrate combustion and EE in mice. However, for all observed traits the distributions turned out to be rather continuous and illustrate that a large number of genes and likely also interactions between genetic regions are shaping opposite phenotypes. Nevertheless, the present catalog of comparative behaviors and responses will help to select opposite phenotypes for activity and metabolic studies and thus enable the discovery of causal genes contributing to the modulation of neural and metabolic pathways.

## Data Availability Statement

Raw data are publicly available through the Mouse Phenome Database from the Jackson Laboratories or can be requested from the authors.

## Ethics Statement

All research and animal care procedures were reviewed by the local animal ethics committee of University of Erlangen, and approved by the local district government (Regierung von Unterfranken) under registry 55.2 2532-2-240. Experiments were conducted in accordance with the Guidelines of the European Parliament Council (directive 2010/62EU). The study conforms to the local as well as ARRIVE (Animal Research Reporting of *in vivo* Experiments) guidelines (Kilkenny et al., 2010).

## Author Contributions

CK, A-CP, and AK acquired data. CK, A-CP, AK, SH, and KZ analyzed the data. CK, IT, and KZ prepared the figures. CK and KZ wrote the draft. All authors contributed to the writing of the final manuscript and approved the final manuscript.

## Conflict of Interest

The authors declare that the research was conducted in the absence of any commercial or financial relationships that could be construed as a potential conflict of interest.

## Footnotes

1 https://phenome.jax.org/

2 http://mousebehavior.org
